# A Diagnostic Challenge in the Emergency Department: Acute Intermittent Porphyria Unmasked After Cabergoline Exposure

**DOI:** 10.7759/cureus.108146

**Published:** 2026-05-02

**Authors:** Christeen Mina, Hady Ghanem, Nancy Matar

**Affiliations:** 1 Emergency Department, Lebanese American University Medical Center, Beirut, LBN; 2 Hematology/Oncology, Lebanese American University, Beirut, LBN

**Keywords:** abdominal pain, acute intermittent porphyria, cabergoline, diagnostic delay, emergency medicine, endocrine triggers, glucose therapy, metabolic disorders, pituitary prolactinoma

## Abstract

Acute intermittent porphyria is a rare metabolic disorder that often presents with recurrent abdominal pain and nonspecific gastrointestinal symptoms, frequently leading to diagnostic delays in emergency settings. We report the case of a 24-year-old woman with glucose-6-phosphate dehydrogenase deficiency diagnosed at birth and pituitary prolactinoma treated with cabergoline 0.5 mg twice weekly, initiated one month before symptom onset, who presented with recurrent severe epigastric pain and vomiting over the course of one year. Repeated laboratory investigations and contrast-enhanced computed tomography of the abdomen and pelvis were unremarkable, and symptoms required opioid analgesia during multiple emergency department visits. A subsequent metabolic evaluation revealed elevated urinary porphyrin precursors, confirming acute intermittent porphyria. Following diagnosis, the cabergoline dose was reduced to 0.25 mg twice weekly with close monitoring of prolactin levels. During two years of follow-up, she experienced two recurrent attacks that were successfully managed with 2 L of 10% dextrose administered intravenously over three hours, along with opioid analgesia. This case highlights the importance of considering acute intermittent porphyria in patients with recurrent unexplained abdominal pain and emphasizes the possible role of medication-related and endocrine factors as indirect triggers, particularly in resource-limited settings where definitive therapy may not be available.

## Introduction

Acute intermittent porphyria is a rare autosomal dominant disorder of heme biosynthesis caused by reduced activity of porphobilinogen deaminase [1]. During acute attacks, increased hepatic heme demand leads to upregulation of delta-aminolevulinic acid synthase, resulting in accumulation of neurotoxic porphyrin precursors, particularly delta-aminolevulinic acid and porphobilinogen. These metabolites are responsible for the predominantly neurovisceral presentation of acute intermittent porphyria, explaining why patients may present with severe gastrointestinal symptoms despite an underlying metabolic disorder.

Acute intermittent porphyria typically presents with neurovisceral attacks characterized by severe abdominal pain, nausea, vomiting, and constipation, and may also be associated with autonomic dysfunction, peripheral neuropathy, seizures, and neuropsychiatric manifestations such as anxiety, confusion, or behavioral changes [2-4]. Routine imaging and laboratory investigations are often normal or nonspecific, contributing to frequent misdiagnosis and significant diagnostic delays, particularly in emergency settings [5,6].

Young women are at the highest risk for acute intermittent porphyria attacks, which are commonly precipitated by medications, hormonal changes, fasting, or metabolic stress [7-9]. Although cabergoline is not known to be a major hepatic enzyme inducer or a recognized high-risk porphyrinogenic medication, it suppresses prolactin through dopamine D2 receptor agonism and may influence gonadal axis function [10]. In susceptible individuals, endocrine shifts and reduced carbohydrate intake are recognized factors that may contribute to acute attacks.

This case highlights a delayed diagnosis of acute intermittent porphyria following nearly one year of recurrent abdominal pain with unremarkable investigations. It also explores a possible temporal association between cabergoline initiation and symptom onset, emphasizing the diagnostic challenges encountered in emergency medicine.

## Case presentation

A 24-year-old woman presented to the emergency department with severe episodic epigastric pain and persistent vomiting. These symptoms had recurred intermittently over a one-year period, during which she had three documented emergency department visits.

Her medical history was significant for glucose-6-phosphate dehydrogenase deficiency diagnosed at birth and a pituitary prolactinoma diagnosed approximately one month before symptom onset, for which she was started on cabergoline 0.5 mg twice weekly. She continued taking cabergoline during the period of recurrent symptoms. She denied tobacco or recreational drug use and reported only occasional alcohol consumption, with no binge drinking preceding the attacks. She was not taking oral contraceptive pills and reported no recent surgery, fasting, major dietary changes, or significant physical or emotional stressors before symptom onset. There was no family history of porphyria.

During each presentation, she appeared in significant distress due to pain but remained hemodynamically stable. Physical examination consistently revealed epigastric tenderness without guarding or peritoneal signs. No neurological deficits, sensory abnormalities, seizures, confusion, or psychiatric features were observed during her emergency department presentations. Vomiting was non-bilious and non-bloody.

Laboratory investigations, including complete blood count, serum electrolytes, renal profile, liver function tests, inflammatory markers, and lipase, were performed and were within normal limits. Pertinent laboratory findings are summarized in Table 1. An incidental borderline reduction in serum sodium was noted on initial laboratory testing (135 mmol/L). Given the absence of clinically significant hyponatremia, no specific treatment or further diagnostic workup was pursued, and subsequent sodium levels remained within the reference range. Urinalysis was unremarkable.

**Table 1:** Laboratoy serum biochemical workup. eGFR, estimated glomerular filtration rate.

Laboratory test	Result	Unit	Reference range
Sodium, serum	135	mEq/L	136-145
Potassium, serum	3.7	mEq/L	3.5-5.1
Chloride, serum	99	mEq/L	98-107
CO_2_, serum	22	mEq/L	22-29
Creatinine, serum	0.51	mg/dL	0.5-1.0
eGFR	157	mL/min/1.73 m²	>60

**Figure 1:**
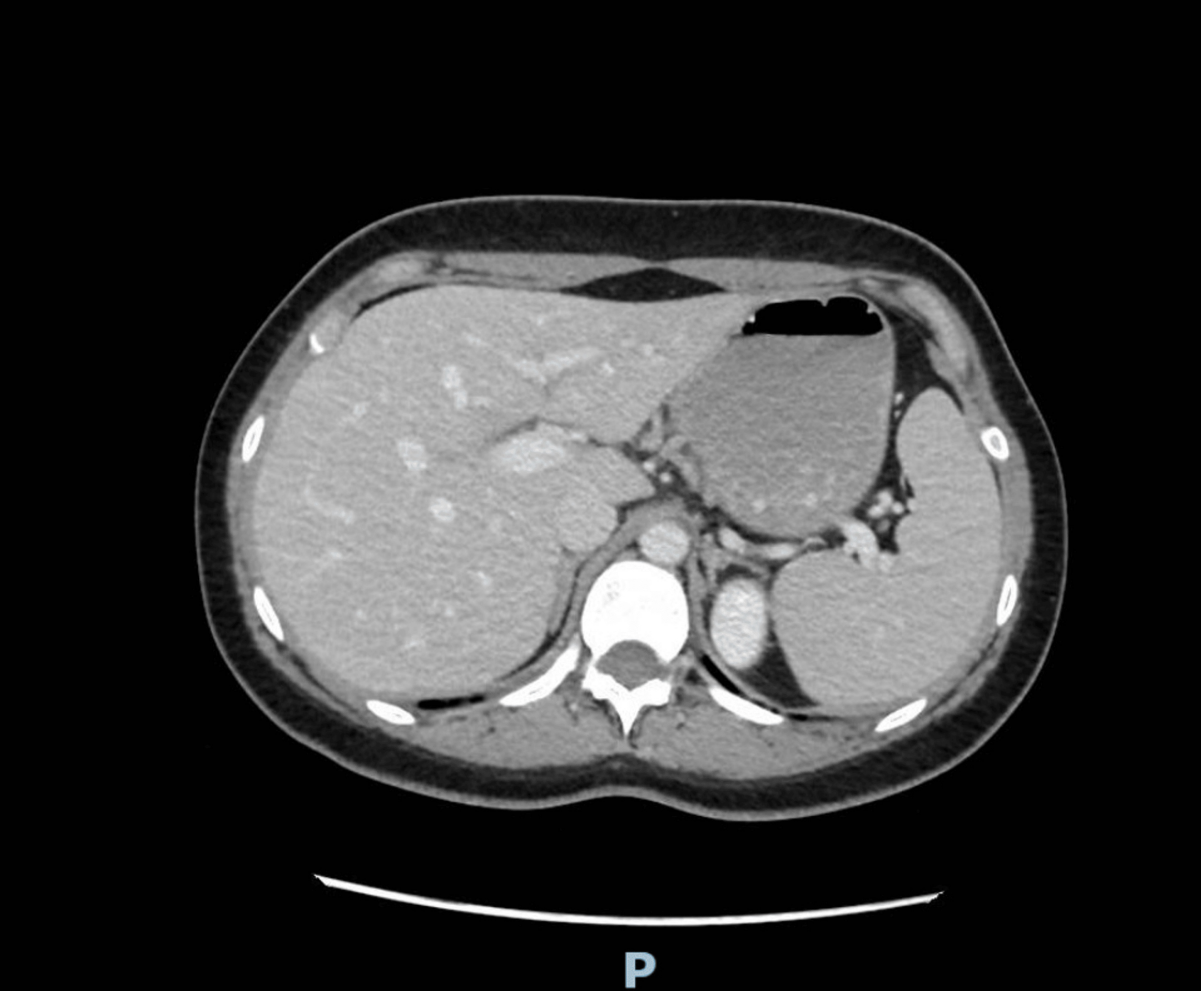
Axial contrast-enhanced CT image of the upper abdomen. No acute hepatobiliary, pancreatic, or upper abdominal abnormality is identified.

**Figure 2:**
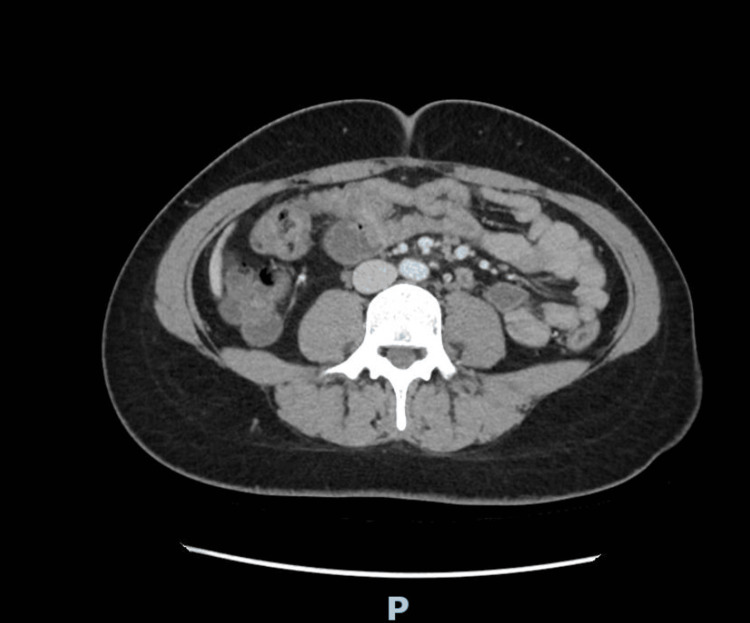
Axial contrast-enhanced CT image of the mid-abdomen. No bowel obstruction, inflammatory process, or intra-abdominal collection is seen.

**Figure 3:**
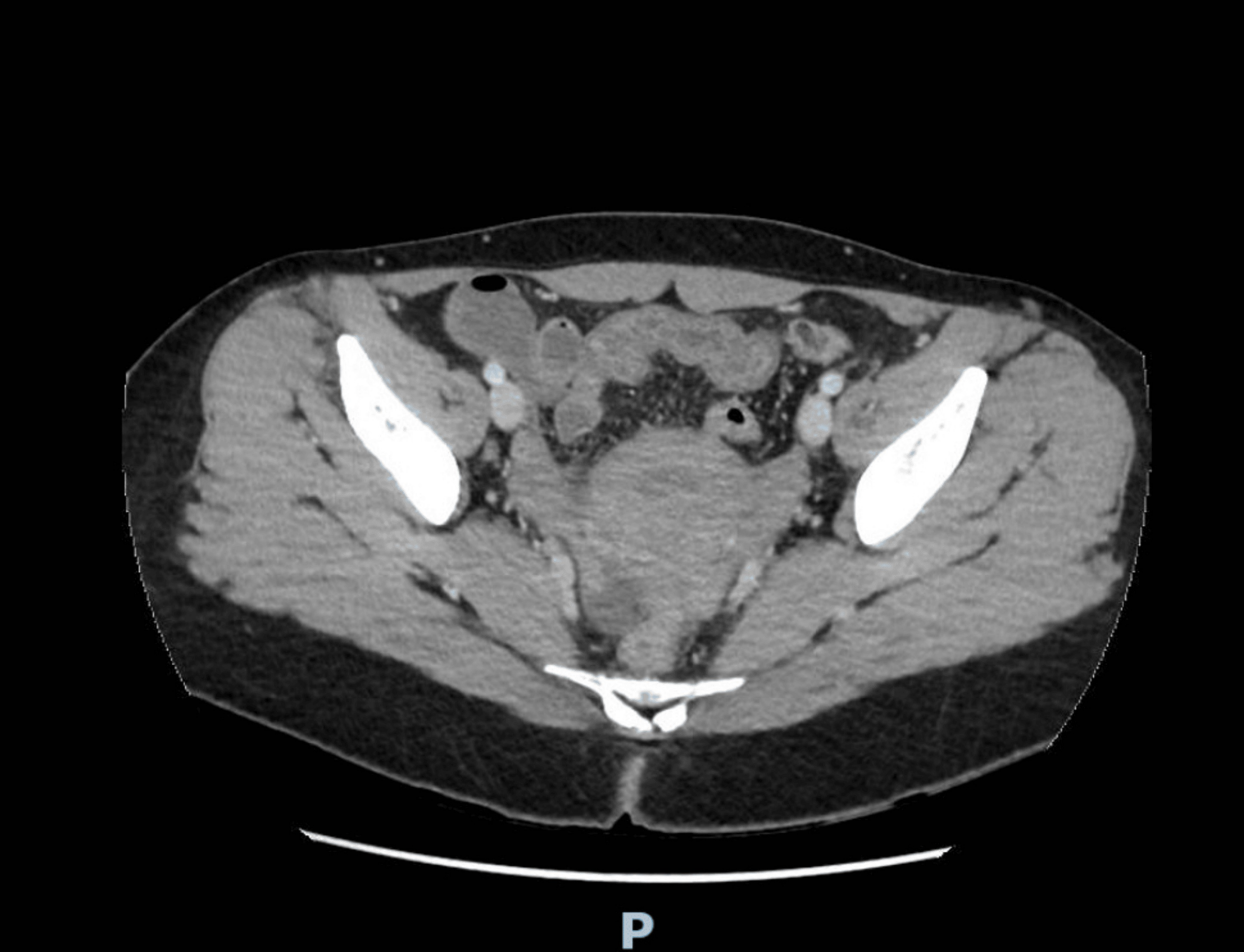
Axial contrast-enhanced CT image of the pelvis. No acute pelvic abnormality or free fluid is identified.

Contrast-enhanced computed tomography of the abdomen and pelvis showed no acute abdominal, pelvic, or hepatobiliary abnormality. Abdominal ultrasonography was not performed; however, liver function tests were within normal limits. Representative contrast-enhanced CT images are provided to document the absence of acute abdominal or pelvic abnormalities despite recurrent severe symptoms (Figures 1-3).

Her pain required intravenous opioid analgesia for partial relief. Due to persistent symptoms and inconclusive findings, a broader metabolic evaluation was conducted in the outpatient setting. Urinary porphyrin analysis of a 24-hour urine collection demonstrated elevated porphyrin precursors, confirming the diagnosis of acute intermittent porphyria. The urine porphyrin analysis is presented in Table 2.

**Table 2:** Urinary porphyrin analysis, 24-hour collection.

Urinary porphyrin analysis	Result	Unit	Reference range
Porphobilinogen	7	µmol/L	Not provided
Porphobilinogen	18	µmol/24 h	<9
Porphobilinogen	4.12	mg/24 h	<2
Uroporphyrins	5	nmol/L	Not provided
Uroporphyrins	13	nmol/24 h	<30
Uroporphyrins	11	µg/24 h	<25
Coproporphyrins I	14	nmol/L	Not provided
Coproporphyrins I	36	nmol/24 h	<38
Coproporphyrins I	24	µg/24 h	<25
Coproporphyrins III	8	nmol/L	Not provided
Coproporphyrins III	21	nmol/24 h	<114
Coproporphyrins III	14	µg/24 h	<75
Total urine volume	2600	mL	Not provided

Following the diagnosis of acute intermittent porphyria, cabergoline was not discontinued because of the need to maintain prolactinoma control; however, the dose was reduced from 0.5 mg to 0.25 mg twice weekly, with close follow-up of prolactin levels. Over a two-year follow-up period, the patient experienced two recurrent attacks, both of which were successfully managed with 2 L of 10% dextrose administered intravenously over three hours, along with opioid analgesia.

## Discussion

Acute intermittent porphyria presents a significant diagnostic challenge because acute attacks commonly manifest with nonspecific neurovisceral symptoms, most frequently severe abdominal pain, nausea, vomiting, constipation, autonomic dysfunction, and, in some cases, neurological or psychiatric manifestations. Routine laboratory and imaging findings may be normal or nonspecific, contributing to repeated misdiagnosis as functional or gastrointestinal disorders and prolonged diagnostic delays, particularly in young women presenting to emergency departments [5,6,11].

Medication and hormonal triggers play a central role in precipitating acute attacks [8,9,12]. Various drug classes, including barbiturates and antiepileptics, are known to induce hepatic delta-aminolevulinic acid synthase, increasing porphyrin precursor production [8,9]. Although cabergoline is not classified as a high-risk porphyrinogenic medication and is not known to be a major hepatic enzyme inducer, dopamine agonists such as cabergoline suppress prolactin through dopamine D2 receptor agonism and may restore gonadal axis function in patients with prolactinomas [10]. In susceptible individuals, endocrine shifts, reduced carbohydrate intake, fasting, and certain medications are recognized precipitants of acute attacks [7-9].

In this case, cabergoline was initiated at 0.5 mg twice weekly approximately one month before symptom onset, and the patient continued therapy throughout the period of recurrent attacks. Other common triggers were not identified, including oral contraceptive use, recent surgery, fasting, major dietary change, binge alcohol intake, or significant physical or emotional stressors. Therefore, we hypothesize that cabergoline may have contributed indirectly through endocrine modulation and/or gastrointestinal adverse effects, leading to reduced carbohydrate intake, rather than through direct hepatic enzyme induction. This proposed association remains inferential and should not be interpreted as proven causality.

This case is notable for the possible temporal association between cabergoline use and the precipitation of recurrent acute intermittent porphyria symptoms, which is not well established in the current literature. After diagnosis, cabergoline was not discontinued because of the need to maintain prolactinoma control, but the dose was reduced to 0.25 mg twice weekly with close monitoring of prolactin levels. During two years of follow-up, the patient experienced two recurrent attacks, both successfully treated with 2 L of 10% dextrose administered intravenously over three hours, along with opioid analgesia, in a setting where hemin therapy was not available.

Although hyponatremia has been described in acute intermittent porphyria, the borderline sodium value in this case was incidental and was not considered clinically significant [13,14].

Acute attacks are typically managed with intravenous hemin, which inhibits hepatic delta-aminolevulinic acid synthase and decreases neurotoxic metabolite production [8,9,15-17]. However, in resource-limited settings where hemin is unavailable, supportive management with intravenous dextrose, adequate analgesia, avoidance of triggers, and close follow-up may provide effective symptom control in selected cases.

## Conclusions

Acute intermittent porphyria should be considered in patients presenting with recurrent unexplained abdominal pain and normal or nonspecific routine investigations, particularly when symptoms recur despite symptomatic treatment. Early recognition of potential medication-related and endocrine triggers is essential to avoid diagnostic delays and unnecessary interventions. This case underscores the importance of maintaining a high index of suspicion in patients with repeated emergency department visits and inconclusive evaluations. Prompt diagnosis, trigger modification, and appropriate supportive management can improve patient outcomes, particularly in settings with limited access to hemin therapy.
